# Clinical concordance between the Gail and Tyrer–Cuzick breast cancer risk models in a Turkish population with high mammographic density prevalence: A cross-sectional study

**DOI:** 10.1097/MD.0000000000048805

**Published:** 2026-05-08

**Authors:** Çağri Akalin

**Affiliations:** a Department of General Surgery, Ordu University Training and Research Hospital, Ordu, Türkiye.

**Keywords:** breast density, breast neoplasms, early detection of cancer, mammography, mass screening, risk assessment, Turkey

## Abstract

The Gail and Tyrer–Cuzick models draw on different input variables and can assign the same woman to different risk categories, yet whether this discordance alters clinical risk stratification in populations with high mammographic density prevalence has not been formally assessed. This study aimed to compare clinical concordance and density-driven risk reclassification between the 2 models in a Turkish screening cohort. In this prospective cross-sectional study, lifetime breast cancer risk was calculated using both models in 1365 women aged 35 to 75 undergoing routine screening at Ordu University Training and Research Hospital from June 2025 to January 2026. Mammographic density was assessed by independent radiologists blinded to clinical data. Concordance at the ≥20% lifetime risk threshold was evaluated using Cohen kappa and the prevalence-adjusted bias-adjusted kappa. The mean age was 50.6 ± 7.8 years; 77.2% had dense breasts (Breast Imaging Reporting and Data System Type C/D). The Gail model classified 40 women (2.9%) as high risk (≥20% lifetime risk); the Tyrer–Cuzick model classified 232 (17.0%; *P* < .001). Cohen κ was 0.22 (95% confidence interval: 0.15–0.29), deflated by marginal asymmetry; the prevalence-adjusted bias-adjusted κ was 0.70, corresponding to substantial agreement (Prevalence Index, 0.80; Bias Index, 0.14; observed agreement, 85.2%). A total of 182 women (13.3%; 95% confidence interval: 11.6–15.3%) classified as low or average risk by Gail were reclassified as high risk by the Tyrer–Cuzick model. The reclassification rates were 14.5% for dense breasts (Type C/D) and 24.1% for extremely dense breasts (Type D). The sensitivity analyses confirmed that mammographic density was the primary driver of inter-model discordance (κ = 0.19; upstaging rate = 10.8%). In this Turkish screening cohort, the Gail and Tyrer–Cuzick models showed only fair concordance at the ≥20% threshold for supplemental magnetic resonance imaging, with mammographic density as the principal driver of disagreement. Outcome-linked validation in populations with high breast density prevalence is needed before either model can be recommended as the reference.

## 1. Introduction

Whether a woman qualifies for supplemental breast magnetic resonance imaging (MRI) screening or chemoprevention depends on her estimated lifetime breast cancer risk.^[1]^ The Gail and Tyrer–Cuzick models are the 2 most widely applied prediction tools in routine practice,^[2,3]^ but they draw on different input variables and can assign the same woman to different risk categories.

Mammographic breast density is relevant to both risk and detection. Dense tissue is an independent predictor of malignancy. Women with extremely dense breasts face a roughly 4- to 6-fold higher risk than those with fatty tissue.^[4,5]^ This density is also a well-documented cause of reduced mammographic sensitivity, as dense parenchyma may mask the development of tumors.^[6,7]^ A meta-analysis in Asian women confirmed that this density–cancer link holds across ethnic groups: risk increased approximately 1.73-fold for each 25% increment in percent density among postmenopausal women.^[8]^ The Gail model, however, does not account for breast density. It relies on age, reproductive history, first-degree family history, and breast biopsy findings.^[2]^ Tice et al^[9]^ showed that adding density to a Gail-type framework improved risk stratification, and several authors have argued that omitting density is a meaningful blind spot in any model intended for populations where dense breasts are common.^[10,11]^

The Tyrer–Cuzick model incorporates a wider array of risk factors. Beyond the variables used by the Gail model, it also accounts for mammographic density, body mass index (BMI), second-degree family history (grandmothers and aunts from both lineages), and hormonal exposures such as menopausal hormone replacement therapy (HRT).^[3]^ Prospective validation cohorts have reported better calibration and discrimination for the Tyrer–Cuzick model than for the Gail model. Terry et al^[12]^ compared 4 models in 18,856 women followed for 10 years; the Tyrer–Cuzick *C*-statistic was 0.71 compared with 0.60 for the Gail model, and the expected-to-observed ratio was near unity for the Tyrer–Cuzick model but 0.79 for the Gail model. Zhang et al^[13]^ found a receiver operating characteristic area of 0.786 for the Tyrer–Cuzick model, compared with 0.665 for the Gail model, in a Chinese case–control study; this difference held outside Western populations. The Tyrer–Cuzick model is not accurate in all subgroups. Valero et al^[14]^ reported a *C*-index of only 0.493 when the Tyrer–Cuzick model was applied to 1192 women with lobular carcinoma in situ, and Wolf et al^[15]^ concluded in a 2024 overview that no single model has gained acceptance across all clinical settings.

The performance of risk prediction models varies across populations. In Spain, the Gail model overestimated the risk of invasive breast cancer in a large validation cohort.^[16]^ A comparable finding was reported in Italy.^[17]^ In both settings, the baseline incidence rates and reproductive patterns differed from the North American reference data.^[16,17]^ Niell et al^[18]^ evaluated 3 models (modified Gail, Tyrer–Cuzick, and BRCAPRO) in 3219 women undergoing screening mammography: Tyrer–Cuzick classified 12.1% as high risk ≥20% lifetime risk (LTR) and the Gail model only 4.4%; just 0.2% were concordant across all 3 tools. Schonberg et al^[19]^ found a kappa value of 0.08 between the Gail and Tyrer–Cuzick models in a primary-care cohort of women in their 40s. Turkey shares several epidemiological characteristics with these Mediterranean populations, namely comparable incidence rates, similar reproductive patterns, and a high prevalence of mammographically dense breast tissue in screening cohorts.^[20]^ Açikgöz and Ergör assessed breast cancer risk using both models in 227 Turkish women from İzmir.^[21]^ However, their study focused on screening compliance rather than inter-model concordance. The authors used different risk time horizons for each model and did not report agreement statistics or density-stratified reclassification. No study has yet compared clinical concordance and density-driven risk reclassification between the 2 models in a Turkish population.

This study aimed to evaluate the clinical concordance between the Gail and Tyrer–Cuzick breast cancer risk models and quantify density-driven risk reclassification in a Turkish screening population with a high prevalence of mammographic density.

## 2. Materials and methods

### 2.1. Study design and participants

This prospective cross-sectional study was conducted at the Breast Diseases Outpatient Clinic of Ordu University Training and Research Hospital between June 2025 and January 2026, with concurrent data collected during routine screening visits. The study cohort comprised 1365 female participants aged 35 to 75 years who presented for routine breast cancer screening. Consecutive eligible women attending the clinic during the study period were invited to participate; no selective sampling was applied beyond the predefined exclusion criteria. A prespecified set of exclusion criteria was applied to ensure an appropriate study population for the comparison of standard population-based risk models. Women were excluded from the analysis if they had a personal history of breast cancer, prior aesthetic or reduction breast surgery, or known pathogenic germline mutations BReast CAncer gene (BRCA) 1/2 documented in medical records. Systematic genetic testing was not performed as part of this study; the BRCA exclusion was based on existing medical record documentation (n = 7 excluded). The study was designed, conducted, and reported in accordance with the Strengthening the Reporting of Observational Studies in Epidemiology guidelines for cross-sectional studies.^[22]^

The age range of 35 to 75 was selected to cover the validated ranges for both models: the Gail model is validated for women aged ≥35, while the Tyrer–Cuzick model has been primarily validated for ages 40 to 70. A total of 14 women aged 35 to 39 fell outside the primary Tyrer–Cuzick validation range; a sensitivity analysis excluding these women did not materially alter the primary findings (κ = 0.22 vs 0.22 in the full cohort).

### 2.2. Ethical considerations

The Clinical Research Ethics Committee of Ordu University approved the study protocol (Decision No: 2025/213; Date: June 20, 2025). All research activities were performed in accordance with the principles of the Declaration of Helsinki. Written informed consent was obtained from all participants prior to participation following a detailed explanation of the study objectives and data collection procedures.

### 2.3. Data collection and risk evaluation models

Individualized breast cancer risk was calculated for each participant using 2 established predictive tools: the Gail model and the Tyrer–Cuzick model. Detailed clinical history, including reproductive and multigenerational family history, was collected through structured in-person interviews conducted by trained research personnel using standardized forms.

Gail model: risk estimates were obtained from the National Cancer Institute’s Breast Cancer Risk Assessment Tool, incorporating age, age at menarche, age at first live birth, number of first-degree female relatives with breast cancer, and number and histological findings of previous breast biopsies.

Tyrer–Cuzick Model (v8.0b): risk estimates were generated with the International Breast Cancer Intervention Study (IBIS) risk evaluator software, version 8.0b (https://ems-trials.org/riskevaluator/). Mammographic breast density, BMI, and an extended family history encompassing second-degree relatives (grandmothers and aunts from both paternal and maternal lineages) are integrated into this tool. Family history was ascertained using a dedicated multigenerational pedigree form that systematically queried both paternal and maternal lineage relatives, including cousins, to maximize ascertainment sensitivity.

Hormonal exposure was captured as a binary “ever-use” variable during the structured interviews. The composite covered menopausal HRT, prolonged oral contraceptive pill (OCP) use beyond age 40, and tibolone. Such a broad definition was chosen to match the input structure required by the IBIS risk calculator, yielding higher prevalence estimates than studies that only report current menopausal HRT use. A more granular classification (i.e., disaggregating HRT from OCP and tibolone) was not feasible for 2 reasons. First, during the structured interviews, participants frequently could not recall the specific name or formulation of the hormonal agent they had used, which is a common limitation in population-based screening settings where women may have discontinued therapy years earlier. Second, verification through the hospital information management system was not possible because the study was conducted in a breast diseases outpatient clinic setting with restricted access to pharmacy dispensing records maintained by external primary-care facilities. It is acknowledged that grouping agents with different risk profiles into a single binary variable may introduce asymmetric measurement error, as hormonal exposure is included in the Tyrer–Cuzick model but not in the Gail model. The potential direction and magnitude of this bias are addressed in Section 4.

### 2.4. Radiological evaluation and breast density classification

Screening mammograms were obtained at the Cancer Early Diagnosis, Screening, and Training Center (KETEM – Kanser Erken Teşhis, Tarama ve Eğitim Merkezi), affiliated with the Ordu Provincial Health Directorate. KETEMs are government-operated population-based screening centers established by the Turkish Ministry of Health to deliver nationwide standardized cancer screening services.^[20]^ Mammographic breast density was assessed on the most recent screening mammogram obtained during the study period. All mammograms were acquired using full-field digital mammography systems. Density was evaluated by experienced breast radiologists at the KETEM, who were institutionally independent of the research team. The KETEM radiologists had no access to the clinical history, family history, or risk model calculations of the participants at the time of the density assessment. Prior mammograms were not available to KETEM radiologists at the time of density assessment. Density was categorized according to the 5th edition of the Breast Imaging Reporting and Data System (BI-RADS)^[23]^: Type A (almost entirely fatty), Type B (scattered areas of fibroglandular density), Type C (heterogeneously dense), and Type D (extremely dense). For risk stratification, the participants were grouped into “Non-Dense” (Type A + B) and “Dense” (Type C + D) categories.

Interobserver reliability was assessed in a random subset of 150 mammograms (approximately 11% of the cohort), each scored independently by 2 breast radiologists blinded to one another’s readings. The weighted Cohen κ for the 4-category BI-RADS density classification was 0.81 (95% confidence interval [CI]: 0.76–0.86), indicating substantial agreement. When the data were collapsed into the binary dense/non-dense classification used in the primary analysis, the agreement was 93.3% (κ = 0.84).

### 2.5. Risk categorization

Participants were categorized into 3 strata of lifetime breast cancer risk (i.e., ascending thresholds of predicted risk over the remaining lifetime): low/average risk (<15% LTR), intermediate risk (15–20% LTR), and high risk (≥20% LTR). The 20% threshold is clinically significant, as it determines eligibility for supplemental screening with breast MRI according to the guidelines of the American Cancer Society.^[24]^

### 2.6. Statistical analysis

The analyses were performed using SPSS v25.0 (IBM Corp.). Normality was checked with the Kolmogorov–Smirnov test, interpreted alongside histograms and *Q*–*Q* plots because, at this sample size, the test flags trivial departures that carry no practical weight. Because both models were applied to the same women, paired methods were used throughout. The mean LTR was compared with a paired *t* test, and the Wilcoxon signed-rank test was used as a nonparametric check. Proportions were compared with chi-square tests, and McNemar test was used for the paired high-risk classification. The within-subject design handles between-subject confounding; therefore, no additional covariate adjustment was performed; estimates are reported unadjusted with 95% CIs.

Inter-model agreement across the 3 risk categories was quantified with Cohen κ (with 95% CI), interpreted as poor (<0.20), fair (0.20–0.40), moderate (0.40–0.60), substantial (0.60–0.80), and almost perfect (>0.80).^[25]^ Because κ is well known to be sensitive to marginal imbalance, the so-called kappa paradox, it was complemented with 3 supplementary indices derived from the binary (≥20% vs <20% LTR) 2 × 2 table: the Prevalence Index (PI = |*a* − *d*|/*N*, with *a* and *d* the concordant cells), the Bias Index (BI = |*b* − *c*|/*N*, with *b* and *c* the discordant cells), and the prevalence-adjusted bias-adjusted kappa (PABAK = 2*P*_0_ − 1, where *P*_0_ is the observed proportion of agreement).^[25,26]^ A high PI deflates κ even when observed agreement is high, whereas a high BI inflates it; reporting PI, BI, and PABAK alongside κ therefore guards against both distortions.

Risk reclassification (upstaging) was defined as a transition from Gail low or average risk (<15% LTR) to Tyrer–Cuzick high risk (≥20% LTR), and the same threshold was applied to the density-stratified subgroups. CIs for mean paired differences were obtained from the paired t-test standard error; CIs for proportions were computed with the Clopper–Pearson exact method. Statistical significance was set at 2-sided *P* < .05. Complete-case analysis was used: 12 women with missing risk-factor data were excluded rather than imputed, because Gail/Breast Cancer Risk Assessment Tool and IBIS require the full input set to run. Two sensitivity analyses were prespecified: exclusion of women aged 35 to 39, who fall outside the primary Tyrer–Cuzick validation range, to test age-range robustness; and restriction to women without a second-degree family history (n = 1117), to separate the effect of mammographic density from extended family history on inter-model discordance.

### 2.7. Sample size justification

The primary outcome was inter-model concordance at the ≥20% lifetime risk threshold, quantified by Cohen κ with the supplementary indices defined above (PI, BI, and PABAK). The cohort-level reclassification rate (Gail low/average → Tyrer–Cuzick high risk) and density-stratified inter-model discordance were designated as secondary outcomes. The sample size was estimated a priori for Cohen kappa using the method of Sim and Wright.^[27]^ With an expected κ = 0.25 (fair agreement, based on published evidence suggesting moderate discordance between these models), a null hypothesis κ_0_ = 0.10, α = 0.05 (2-sided), and statistical power = 0.90, the minimum required sample size was estimated at approximately 1100 participants. A total of 1365 women were enrolled, providing a 24% margin above the minimum required sample size to account for potential incomplete data and support subgroup analyses by breast density category.

### 2.8. Assessment and reduction of potential bias

Several potential bias sources were identified and addressed in the study design. Selection bias: The study was conducted at a university-based breast disease clinic that functions as both a primary screening facility and a referral center for the Ordu province. Although all included participants presented for routine screening rather than diagnostic evaluation, the clinic setting may be enriched for women with increased breast awareness or known risk factors, which may limit the generalizability of the results to the larger population. Information bias: self-reported reproductive and family history data are subject to recall bias. To mitigate this risk, all data were collected through structured interviewer-administered pedigree forms; this approach has been shown to improve the accuracy of family history ascertainment compared with self-administered questionnaires. Observer bias: mammographic density was assessed by KETEM radiologists who were institutionally independent of the research team and had no access to clinical or family history data, thereby providing structural blinding at the institutional level. Inter-rater reliability was substantial (weighted κ = 0.81; see Section 2.4).

## 3. Results

### 3.1. Participant flow and demographic characteristics

**Figure 1.**
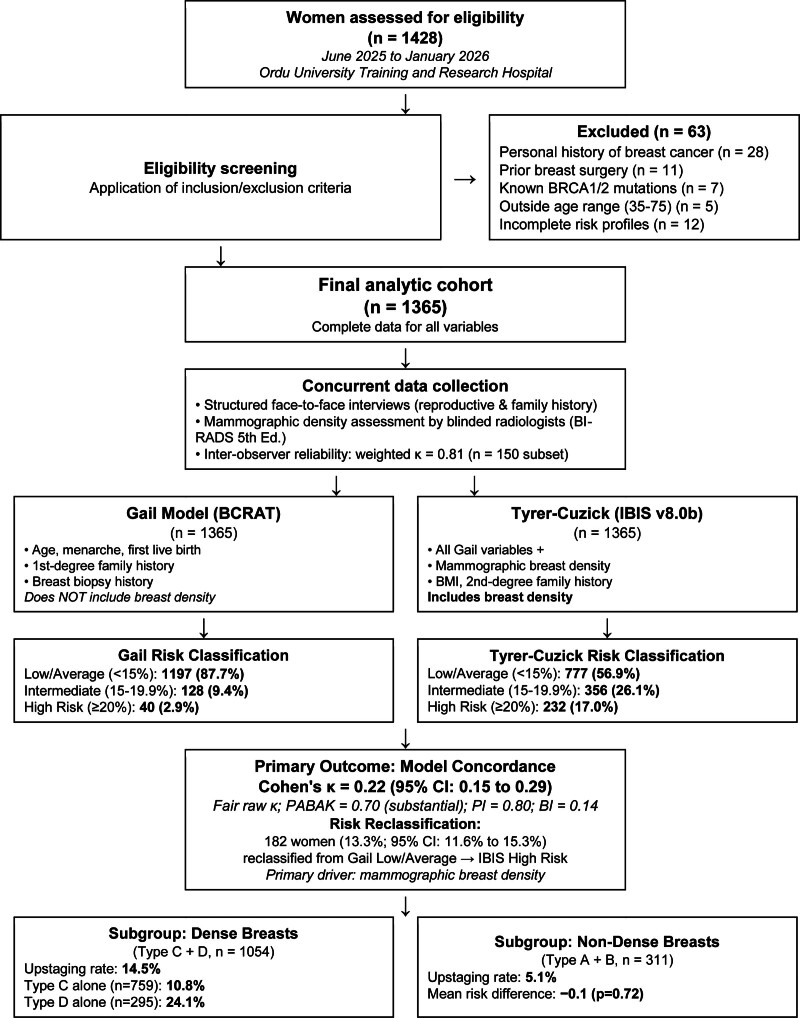
Flow diagram of participant selection, including the number of women assessed for eligibility (n = 1428), reasons for exclusion, and the final analytic cohort (n = 1365).

Between June 2025 and January 2026, 1428 women were initially assessed for eligibility. Of these, 63 were excluded: 28 had a personal history of breast cancer, 11 had prior breast surgery (aesthetic or reduction), 7 had known pathogenic BRCA1/2 mutations documented in their medical records, 5 were outside the eligible age range (35–75 years), and 12 had incomplete risk-factor profiles preventing calculation of both risk models. A total of 1365 women with complete data for all variables required by both models were evaluated in the final analysis. Figure 1 shows the participant flow.

The baseline demographic and clinical characteristics of the cohort are summarized in Table 1. The mean age was 50.6 ± 7.8 years (range: 35–75), and the mean BMI was 28.6 ± 5.0 kg/m^2^. Approximately 51.3% of the women were postmenopausal. The mean age at menarche was 13.1 ± 1.5 years, and the mean parity was 2.6 ± 1.2, with only 3.5% nulliparous.

**Table 1:** Baseline demographic and clinical characteristics.

Variable	Value or n (%)
Age (yr), mean ± SD	50.6 ± 7.8
35–44	285 (20.9%)
45–54	656 (48.1%)
55–64	327 (24.0%)
≥65	97 (7.1%)
Body mass index (kg/m^2^), mean ± SD	28.6 ± 5.0
<25 (normal)	300 (22.0%)
25–29.9 (overweight)	517 (37.9%)
≥30 (obese)	548 (40.1%)
Menopausal status	
Premenopausal	665 (48.7%)
Postmenopausal	700 (51.3%)
Age at menarche (yr), mean ± SD	13.1 ± 1.5
Parity, mean ± SD	2.6 ± 1.2
Nulliparous	48 (3.5%)
First-degree family history of breast cancer	129 (9.5%)
Second-degree family history of breast cancer	249 (18.2%)
Previous breast biopsy	215 (15.8%)
Atypical ductal hyperplasia	16 (1.2%)
Hormonal exposure (ever use)	441 (32.3%)
Mammographic breast density (BI-RADS)	
Type A (fatty)	59 (4.3%)
Type B (scattered)	252 (18.5%)
Type C (heterogeneously dense)	759 (55.6%)
Type D (extremely dense)	295 (21.6%)
Dense breasts (C + D)	1054 (77.2%)

Total n = 1365. Data are presented as n (%) or mean ± SD unless otherwise specified.

BI-RADS = Breast Imaging Reporting and Data System; SD = standard deviation.

A first-degree family history of breast cancer was present in 9.5% of the participants, while extended family history (grandmothers, aunts, or cousins) was reported by 18.2%. A previous breast biopsy was documented in 15.8%, and atypical ductal hyperplasia was present in 1.2%. Exogenous hormonal exposure (ever-use of HRT, prolonged OCP, or tibolone) was reported by 32.3% of the participants.

### 3.2. Mammographic breast density distribution

The mammographic breast density distribution according to BI-RADS classification was as follows: Type A (fatty): 59 (4.3%), Type B (scattered): 252 (18.5%), Type C (heterogeneously dense): 759 (55.6%), Type D (extremely dense): 295 (21.6%). In total, 77.2% (n = 1054) of the women had dense breasts (Type C + D). The density distribution data are presented in Table 1.

### 3.3. Risk assessment results

The risk estimates obtained from the Gail and Tyrer–Cuzick models differed substantially (Table 2). The Gail model yielded a mean LTR of 11.2% ± 3.8% (median: 10.4%; interquartile range [IQR]: 8.5–13.2%), whereas the Tyrer–Cuzick model yielded a mean LTR of 14.8% ± 5.6% (median: 13.5%; interquartile range: 10.8–17.9%). The mean difference between the models (Tyrer–Cuzick minus Gail) was 3.6 percentage points (95% CI: 3.2–4.0%), representing a statistically significant difference (paired *t* test: *P* < .001; Wilcoxon signed-rank test: *P* < .001). The mean difference was driven almost entirely by the dense breast subgroup (Type C/D: +4.7 percentage points, *P* < .001), whereas the risk estimates converged in women with non-dense breasts (Type A/B: −0.1 percentage points, *P* = .72).

**Table 2:** Comparison of breast cancer risk estimates between the Gail and Tyrer–Cuzick models.

Parameter	Gail model	Tyrer–Cuzick model	*P*-value
Lifetime risk (%), mean ± SD	11.2 ± 3.8	14.8 ± 5.6	<.001
Lifetime risk (%), median (IQR)	10.4 (8.5–13.2)	13.5 (10.8–17.9)	<.001
Risk categories, n (%)			
High risk (≥20%)	40 (2.9%)	232 (17.0%)	<.001
Intermediate (15–19.9%)	128 (9.4%)	356 (26.1%)	<.001
Low or average (<15%)	1197 (87.7%)	777 (56.9%)	<.001

Data are presented as mean ± SD, median (IQR), or n (%).

*P*-values: paired *t* test (means), Wilcoxon signed-rank test (medians), McNemar test (categories).

IQR = interquartile range; SD = standard deviation.

### 3.4. Risk category distribution

The distribution of risk categories for both models is presented in Table 2. The Gail model classified 87.7% as “low/average risk” (<15% LTR) and identified only 40 women (2.9%; 95% CI: 2.1–4.0%) as “high risk” (≥20%). By comparison, the Tyrer–Cuzick model classified 232 women (17.0%; 95% CI: 15.1–19.1%) as “high risk.” The Tyrer–Cuzick model identified approximately 5.9 times more women as high risk than the Gail model (17.0% vs 2.9%; McNemar test: *P* < .001).

### 3.5. Clinical concordance between the models

The concordance between the Gail and Tyrer–Cuzick risk categories was assessed using Cohen kappa coefficient for the 3-category classification: κ = 0.22 (95% CI: 0.15–0.29), indicating fair agreement. To aid clinical interpretation and address kappa’s known sensitivity to marginal asymmetry, supplementary agreement indices were computed from the binary classification (≥20% vs <20%). The observed proportion of agreement between the 2 models was 85.2%. The PI was 0.80, and the BI was 0.14. Given the high PI, reflecting that the overwhelming majority of women were classified as non-high risk by both models, the raw kappa value is expected to be deflated relative to the true level of agreement.^[25]^ The PABAK, which corrects for both prevalence asymmetry and systematic rater bias, was 0.70, corresponding to what would conventionally be termed substantial agreement.^[26]^ While the 2 models assign discordant risk categories more often than chance, the absolute level of observed concordance is considerably higher than the raw kappa alone would suggest.

### 3.6. Risk reclassification analysis

The cross-classification of risk categories between models is presented in Table 3. Among the 1197 women classified as “low/average risk” (<15%) by the Gail model: 182 (15.2%) were reclassified as “high risk” (≥20%) by the Tyrer–Cuzick model, 296 (24.7%) were reclassified as “intermediate risk” (15–20%) by the Tyrer–Cuzick model, and 719 (60.1%) remained in the “low/average” category in both models. The overall upstaging rate (Gail low/average → Tyrer–Cuzick high risk) was 13.3% (95% CI: 11.6–15.3%; n = 182) of the entire cohort.

**Table 3:** Risk reclassification matrix between the Gail and Tyrer–Cuzick models.

Gail model category	Tyrer–Cuzick low or average, n (%)	Tyrer–Cuzick intermediate, n (%)	Tyrer–Cuzick high risk, n (%)	Total
Low or average (<15%)	719 (60.1)	296 (24.7)	182 (15.2)	1197
Intermediate (15–19.9%)	53 (41.4)	60 (46.9)	15 (11.7)	128
High risk (≥20%)	5 (12.5)	0 (0.0)	35 (87.5)	40
Total	777 (56.9)	356 (26.1)	232 (17.0)	1365

Data are presented as n (row %).

Cohen κ = 0.22 (95% CI, 0.15–0.29); PI = 0.80; BI = 0.14; PABAK = 0.70; observed agreement = 85.2%. Upstaging rate (Gail low or average → Tyrer–Cuzick high risk): 182/1365 = 13.3% (95% CI, 11.6%–15.3%).

BI = Bias Index; CI = confidence interval; PABAK = prevalence-adjusted bias-adjusted kappa; PI = Prevalence Index.

### 3.7. Subgroup analysis by breast density

The risk estimates stratified by mammographic breast density category are presented in Table 4. Among women with dense breasts (Type C + D, n = 1054): the Gail mean LTR was 11.1% ± 3.6% versus the Tyrer–Cuzick mean LTR of 15.8% ± 5.4% (mean difference: 4.7 percentage points; 95% CI: 4.4–5.0; *P* < .001). The Gail model classified 22 (2.1%) as high risk, compared with 209 (19.8%) by the Tyrer–Cuzick model. The upstaging rate among women with dense breasts was 14.5% (95% CI: 12.4–16.8%; n = 153). Within the dense subgroup, the upstaging rate scaled with density, from 10.8% in Type C (82/759) to 24.1% in Type D (71/295); the same dose-response pattern appears in the underlying mean risk differences (Table 4).

**Table 4:** Risk assessment stratified by mammographic breast density.

Breast density	n (%)	Gail LTR, mean ± SD	Tyrer–Cuzick LTR, mean ± SD	Gail high risk, n (%)	Tyrer–Cuzick high risk, n (%)	Upstaging, n (%)
Type A (fatty)	59 (4.3)	11.8 ± 4.5	10.2 ± 4.2	3 (5.1)	4 (6.8)	1 (1.7)
Type B (scattered)	252 (18.5)	11.4 ± 4.1	11.8 ± 4.9	15 (6.0)	19 (7.5)	15 (6.0)
Type C (heterogeneously dense)	759 (55.6)	11.1 ± 3.5	15.2 ± 5.1	17 (2.2)	125 (16.5)	82 (10.8)
Type D (extremely dense)	295 (21.6)	11.0 ± 3.8	17.8 ± 5.8	5 (1.7)	84 (28.5)	71 (24.1)
Dense (C + D)	1054 (77.2)	11.1 ± 3.6	15.8 ± 5.4	22 (2.1)	209 (19.8)	153 (14.5)
Non-dense (A + B)	311 (22.8)	11.5 ± 4.2	11.4 ± 4.8	18 (5.8)	23 (7.4)	16 (5.1)

Data are presented as mean ± SD or n (%).

High risk: ≥20% lifetime risk. Upstaging: reclassification from Gail non-high-risk to Tyrer–Cuzick high risk within each density stratum.

All *P*-values < .001 for dense vs non-dense comparisons (paired t-test and McNemar test).

LTR = lifetime risk; SD = standard deviation.

Among women with non-dense breasts (Type A + B, n = 311): the Gail mean LTR was 11.5% ± 4.2% versus the Tyrer–Cuzick mean LTR of 11.4% ± 4.8% (mean difference: −0.1 percentage points; 95% CI: −0.6 to 0.4; *P* = .72). The upstaging rate was 5.1% (95% CI: 3.1–8.2%; n = 16). The Type A (fatty) stratum alone showed a reversed pattern, with Tyrer–Cuzick estimates falling below Gail (10.2% ± 4.2 vs 11.8% ± 4.5; n = 59), since low density enters the Tyrer–Cuzick algorithm as a protective factor (Table 4).

Among women with extremely dense breasts (Type D, n = 295), the Tyrer–Cuzick model classified 84 (28.5%) as high-risk versus only 5 (1.7%) by Gail. Of these 84 Tyrer–Cuzick high-risk women, 5 were concordantly classified as high risk by the Gail model, 71 were upstaged from the Gail low or average category (consistent with the global upstaging definition), and 8 were reclassified from the Gail intermediate (see Table S1, Supplemental Digital Content). Applying the same upstaging definition used at the cohort level, the Type D upstaging rate was 24.1% (71/295; 95% CI: 19.3–29.3%). The mean risk difference in this subgroup was 6.8 percentage points (Tyrer–Cuzick 17.8 ± 5.8% vs Gail 11.0 ± 3.8%; 95% CI: 6.2–7.4; *P* < .001).

### 3.8. Sensitivity analysis

A sensitivity analysis was conducted among the 1117 women without second-degree family history to assess whether mammographic density or extended family history was the primary driver of model discordance. In this subgroup, the Tyrer–Cuzick model classified 14.1% as high risk, compared with 3.2% by Gail (κ = 0.19; 95% CI: 0.12–0.26), with an upstaging rate of 10.8% (95% CI: 9.0–12.9%). A separate sensitivity analysis excluding 14 women aged 35 to 39 who fell outside the primary Tyrer–Cuzick validation range (40–70 years) did not materially alter the primary findings (κ = 0.22 vs 0.22 in the full cohort; upstaging rate 13.4% vs 13.3%).

## 4. Discussion

### 4.1. Clinical significance of risk reclassification

In this cohort, the 2 models assigned markedly different risk classifications to the same women. The Tyrer–Cuzick model classified 232 women (17.0%) as high risk (≥20% LTR), whereas the Gail model classified 40 (2.9%), a 5.9-fold discrepancy. Within the total cohort, 182 women (13.3%; 95% CI: 11.6–15.3%), corresponding to 15.2% of the 1197 women classified as low or average risk by the Gail model, crossed the ≥20% threshold when the Tyrer–Cuzick model was applied. Had the Tyrer–Cuzick classification been used for clinical decision-making, these 182 women would have met the American Cancer Society threshold for supplemental MRI screening;^[24]^ under the Gail model alone, they would not. This study compared model classification outputs and did not validate either model against cancer outcomes. The discordance shows that the 2 tools yield different clinical assessments, but it does not establish which assessment is more accurate. The higher Tyrer–Cuzick estimates likely reflect the additional input variables (mammographic density, BMI, second-degree family history, and hormonal exposure) that the Tyrer–Cuzick model captures and the Gail model does not. Only prospective outcome data can determine whether this wider classification reflects improved sensitivity or reduced specificity.

The density-stratified analysis confirmed a gradient. Among women with dense breasts (Type C/D), the upstaging rate was 14.5%, compared with 5.1% in women with non-dense tissue (Type A/B). In women whose mammograms showed extremely dense tissue (BI-RADS Type D), the Tyrer–Cuzick model classified 28.5% as high risk, compared with only 1.7% by the Gail model, resulting in an upstaging rate of 24.1%. In women with almost entirely fatty breasts (Type A), the Tyrer–Cuzick model produced lower mean LTR estimates than the Gail model (10.2% vs 11.8%); low mammographic density, in the Tyrer–Cuzick algorithm, functions as a protective factor. In this cohort, breast density (rather than family history or hormonal exposure) was the main source of disagreement between the 2 models. Bae and Kim^[8]^ reported in a meta-analysis that breast cancer risk among Asian women increased approximately 1.73-fold for each 25% increment in percent density, with a stronger association in premenopausal women (summary effect size 3.23; 95% CI: 2.23–4.66) than in postmenopausal women (summary effect size 1.62; 95% CI: 1.13–2.32). That the Tyrer–Cuzick model incorporates this variable while the Gail model does not constitute a structural difference with direct clinical consequences. It should be noted, however, that a higher classification rate is not the same as greater accuracy. Valero et al^[14]^ found at Memorial Sloan Kettering that the Tyrer–Cuzick model achieved a *C*-index of only 0.493 in 1192 women with lobular carcinoma in situ, no better than chance, even though the model assigned a median 10-year risk score above 20%. Without outcome-based verification, it cannot be claimed that a Tyrer–Cuzick high-risk label is more accurate than a Gail low-risk label.

### 4.2. Comparison with existing literature and the kappa paradox

Inter-model discordance in breast cancer risk prediction is not unique to this cohort. Niell et al^[18]^ evaluated 3 models (modified Gail, Tyrer–Cuzick version 7, and BRCAPRO) among 3219 women presenting for screening mammography and found that the Tyrer–Cuzick model classified 12.1% as high risk compared with 4.4% by the Gail model, with women 6.4-fold more likely to be classified as high risk by Tyrer–Cuzick (95% CI: 4.7–8.7); only 6 women (0.2%) were classified as high risk by all 3 tools. This 6.4-fold figure in Niell U.S. cohort closely parallels the 5.9-fold Tyrer–Cuzick-to-Gail ratio observed in this cohort. Schonberg et al.^[19]^ reported a kappa of 0.08 between the 2 models for LTR at the ≥20% threshold in a primary-care cohort of women aged 40 to 49; this was lower than the κ = 0.22 in this cohort. Zhang et al^[13]^ found that the Tyrer–Cuzick model had a receiver operating characteristic area under the curve of 0.786, compared with 0.665 for the Gail model, in a Chinese case–control study, suggesting better discriminatory capacity in an East Asian population. Vianna et al^[28]^ compared the 2 models in 1738 Brazilian women screened in primary care and found that discordance increased as risk estimates rose, with the Gail model consistently underestimating risk compared with the Tyrer–Cuzick model in women with a positive family history. Across North American, European, South American, and Asian cohorts, the pattern holds: inter-model discordance reflects the different input variables the 2 models use, not a feature peculiar to any one population.

The kappa value in this study must be read in context. Cohen kappa is sensitive to the marginal prevalence of the categories being compared; Nelson and Edwards^[25]^ termed this the “kappa paradox.” In the data presented here, the PI for the binary high-risk classification was 0.80. According to both models, most women were non-high risk, and this extreme cell imbalance deflates kappa relative to the actual agreement; the observed concordance of 85.2% corresponds to a raw kappa of only 0.22. The PABAK, which corrects for both prevalence asymmetry and systematic rater bias, was 0.70, indicating substantial agreement.^[26]^ The BI was 0.14, confirming a moderate systematic tendency of the Tyrer–Cuzick model to classify more women as high risk. The 182 women who crossed the ≥20% threshold remained clinically relevant regardless of the agreement metric used. The supplementary indices showed that a raw kappa of 0.22 exaggerated the true level of disagreement.

Validation studies in Mediterranean populations assessed the Gail model against cancer outcomes rather than inter-model agreement, but the direction of their findings is relevant to this analysis. Pastor-Barriuso et al^[16]^ found that the Gail model overestimated the incidence of invasive breast cancer in Spanish women by 46% (expected-to-observed ratio 1.46); Boyle et al^[17]^ reported comparable overestimation in Italy. The Gail model, then, does not perform uniformly across populations, which is consistent with the observed inter-model discordance. Terry et al^[12]^ compared 4 risk models in 18,856 women followed for 10 years and found *C*-statistics of 0.71 for the Tyrer–Cuzick model compared with 0.60 for the Gail model; Glynn et al^[29]^ reported more modest differences (0.63 compared with 0.61) in the Nurses’ Health Study. Meadows et al^[30]^ examined a racially diverse community-based sample and found that among women meeting the ≥20% threshold, 73% were classified by the Tyrer–Cuzick model alone, fewer than 1% by the Gail model alone, and only 4% by both tools; this distribution resembles the 2.9% versus 17.0% split in this cohort. In settings where risk thresholds govern access to supplemental screening, the 2 models cannot be treated as interchangeable.

### 4.3. Clinical implications and future directions

Model selection directly affects who qualifies for supplemental screening. In this cohort, 40 women met the ≥20% MRI eligibility threshold under the Gail model. Under the Tyrer–Cuzick model, this rose to 232, an additional 192 women from a single screening cohort. These additional women could be offered supplemental MRI and chemoprevention, but without outcome data, it is unclear whether the benefits of expanded screening would outweigh the resource burden and the psychological costs of false-positive results. The DENSE trial^[31]^ showed that supplemental MRI in women with extremely dense breast tissue and normal mammography reduced interval cancers by approximately half (2.5 vs 5.0 per 1000 screenings; *P* < .001); however, that trial enrolled exclusively Dutch women aged 50–75 with BI-RADS Type D breasts. Whether Tyrer–Cuzick-identified high-risk women in the current population would see a comparable reduction remains to be determined, especially when their density profile falls outside the narrow Type D subgroup in which MRI benefit was demonstrated.

In Turkey, where risk-based screening policies are still being developed, these data support the adaptation of risk thresholds to local population characteristics rather than the application of Western cutoffs without modification. The decision about which model or combination of models to use for supplemental screening may well depend on the local epidemiological profiles and available resources. Outcome-based validation in Turkish and comparable populations should be considered a prerequisite before any model can be recommended for routine clinical use.

Beyond conventional risk models, newer technologies may further refine stratification. Deep learning-based approaches such as Mirai^[32]^ extract quantitative imaging features from mammograms, and Collister et al^[33]^ showed in the UK Biobank that adding a polygenic risk score to the Tyrer–Cuzick and Gail models raised the Harrell C from 0.57 and 0.54, respectively, to 0.67. Integrating genomic, imaging-based, and clinical risk factors may represent the next step toward more accurate individual-level prediction, although the infrastructure required for such integration is not yet available in most clinical settings.

### 4.4. Strengths and limitations

This study has several strengths. A prospective cohort of 1365 women with complete data for all model variables was assembled. Density was assessed by blinded KETEM radiologists with substantial inter-rater agreement (weighted κ = 0.81). All clinical information was obtained through face-to-face interviews using multigenerational pedigree forms, a method shown to improve family history ascertainment over self-administered questionnaires. To the author’s knowledge, this is the first direct comparison of the Gail and Tyrer–Cuzick models in a Turkish population.

Several limitations require acknowledgments. First, the single-center design at a university breast diseases clinic may limit generalizability. The clinic serves both screening and referral functions, which may have concentrated the cohort toward women with known risk factors, potentially explaining the relatively high prevalence of prior biopsy (15.8%) and second-degree family history (18.2%). Both models were applied to the same women under identical conditions. Referral-based enrichment would inflate risk estimates in both models, but its effect on inter-model agreement is difficult to predict given the differing input structures. The relatively high prior-biopsy prevalence of 15.8% is consistent with this dual screening-referral remit and exceeds what would be expected in pure population-based screening cohorts. The reclassification figures reported here therefore apply most directly to clinic-based mixed screening settings; extrapolation to population-based screening programs will require external validation in a dedicated cohort.

Second, the study compared model outputs without validating either tool against cancer outcomes. Without prospective follow-up data, it is not possible to determine which model is more accurate in this population.

Third, hormonal exposure was defined broadly (ever-use of HRT, prolonged OCP, or tibolone; 32.3%) and enters the Tyrer–Cuzick model but has no equivalent input in the Gail model. Many participants could not recall which hormonal agent they had used during the face-to-face interviews. This is a recognized problem in population-based screening settings, where women may have stopped therapy years earlier. Verification through pharmacy records was not feasible because the study was conducted in an outpatient clinic with restricted access to dispensing data held by external primary-care facilities. Therefore, agent-specific sensitivity analysis (e.g., restricting the comparison to menopausal HRT users only) was not possible. If some women classified as ever-users had taken only short-term oral contraceptives (which carry a smaller breast cancer risk than prolonged HRT), the Tyrer–Cuzick model may have overestimated risk, and the upstaging rate may have been exaggerated. The persistence of discordance in the sensitivity analysis excluding women with second-degree family history (κ = 0.19; upstaging rate 10.8%) identifies mammographic density as the dominant source of inter-model disagreement, although not the only one. The 19% relative reduction in the upstaging rate when extended family history was removed (13.3% → 10.8%) points to a genuine but smaller contribution from that variable.

Fourth, the interpretation of Cohen kappa in this study is complicated by the kappa paradox. As reported in §3.5, the PI was 0.80, which deflates kappa even when the observed agreement is high.^[25]^ The PABAK of 0.70 provides a more balanced picture, and the degree of model agreement, while clinically imperfect, is higher than the raw kappa of 0.22 alone would indicate.^[26]^ The Tyrer–Cuzick model’s tendency toward higher risk estimates does not, by itself, guarantee greater accuracy. In a validation cohort of 132,139 women, Brentnall and Cuzick^[34]^ reported over-prediction in the highest risk decile (observed-to-expected ratio 0.78; 95% CI: 0.69–0.88) and a 2-fold overestimation of short-term risk in the first year after a negative mammographic screen (O/E = 0.49): a “clearance effect” attributable to the model’s constant baseline hazard assumption rather than a population-specific miscalibration.

Fifth, systematic genetic testing was not performed; 7 women were excluded because of documented BRCA mutations, but unidentified carriers may remain in the cohort. Sixth, family history and reproductive data were self-reported and therefore subject to recall bias; structured interview forms with multigenerational pedigree assessment were used to mitigate this source of error, though they cannot eliminate it. The prevalence of density in this cohort (77.2%, BI-RADS Type C/D) also warrants consideration. Dundar et al^[20]^ noted that Turkish population-based screening programs draw participants with varying levels of breast health awareness; the figure at this university clinic may reflect both the younger mean age (50.6 years) and possible self-selection by women with higher awareness. In international screening data, 40% to 50% of women aged 40 to 74 have dense breasts. The higher proportion in this cohort likely reflects the younger age distribution and may limit the generalizability of the absolute reclassification rates to older populations.

## 5. Conclusion

In this Turkish screening cohort of 1365 women, the Gail and Tyrer–Cuzick models showed only fair agreement (κ = 0.22), and model choice materially altered which women met the ≥20% threshold for supplemental MRI screening. Mammographic density, present in more than 3 quarters of the cohort, was the principal driver of this discordance. The performance of both models was developed and validated in general Caucasian populations, and their applicability in settings with high mammographic density prevalence remains under-explored. These models require independent prospective validation against cancer outcomes in non-Western cohorts before either can be recommended as the reference for risk-based supplemental screening.

## Acknowledgments

The author acknowledges the healthcare professionals at Ordu KETEM for their support during data collection and the intern medical doctors who assisted with patient interviews and completion of study forms. The statistical analysis plan was specified a priori, and all analyses were conducted by the author, who has formal training in biostatistics and clinical research methodology. Generative artificial intelligence tools (OpenAI, Claude [Anthropic], Grammarly) were used for language editing, paraphrasing to improve clarity, and assistance with figure layouts. These tools were not used for data generation, statistical analysis, result interpretation, or the formulation of scientific conclusions. The author retains full responsibility for the content of the manuscript.

## Author contributions

**Conceptualization:** Çağri Akalin.

**Data curation:** Çağri Akalin.

**Formal analysis:** Çağri Akalin.

**Funding acquisition:** Çağri Akalin.

**Investigation:** Çağri Akalin.

**Methodology:** Çağri Akalin.

**Project administration:** Çağri Akalin.

**Resources:** Çağri Akalin.

**Software:** Çağri Akalin.

**Supervision:** Çağri Akalin.

**Validation:** Çağri Akalin.

**Visualization:** Çağri Akalin.

**Writing – original draft:** Çağri Akalin.

**Writing – review & editing:** Çağri Akalin.


