# Supplementary material for: Clinical concordance between the Gail and Tyrer–Cuzick breast cancer risk models in a Turkish population with high mammographic density prevalence: A cross-sectional study
Source: Medicine (Baltimore). 2026 May 8;105(19):e48805. doi: 10.1097/MD.0000000000048805 (PMC13166859; doi:10.1097/MD.0000000000048805)
Supplement: Supplementary file 1 [file medi-105-e48805-s001.docx]

**Supplementary Table S1. Cross-Classification of Risk Categories in Women with Extremely Dense Breasts**

| **Gail Category (Type D)** | **Tyrer-Cuzick Low or Average,**  **n (%)** | **Tyrer-Cuzick Intermediate,**  **n (%)** | **Tyrer-Cuzick High Risk,**  **n (%)** | **Total** |
| --- | --- | --- | --- | --- |
| Low or average (<15%) | 115 (41.4) | 96 (34.5) | 71 (25.5) | 282 |
| Intermediate  (15-19.9%) | 0 (0.0) | 0 (0.0) | 8 (100) | 8 |
| High risk  (≥20%) | 0 (0.0) | 0 (0.0) | 5 (100) | 5 |
| **Total** | 115 (39.0) | 96 (32.5) | 84 (28.5) | 295 |
| Data are presented as n (row %). Subgroup refers to women with extremely dense breasts on mammography (Breast Imaging Reporting and Data System Type D; n = 295). Type D upstaging rate (Gail low or average → Tyrer-Cuzick high risk): 71/295 = 24.1% (95% CI, 19.3%–29.3%). An additional 8 women were reclassified from Gail intermediate to Tyrer-Cuzick high risk; combining both categories yields 79/295 = 26.8%. CI = confidence interval. | | | | |
